# Elemental Dynamics in Hair Accurately Predict Future Autism Spectrum Disorder Diagnosis: An International Multi-Center Study

**DOI:** 10.3390/jcm11237154

**Published:** 2022-12-01

**Authors:** Christine Austin, Paul Curtin, Manish Arora, Abraham Reichenberg, Austen Curtin, Miyuki Iwai-Shimada, Robert O. Wright, Rosalind J. Wright, Karl Lundin Remnelius, Johan Isaksson, Sven Bölte, Shoji F. Nakayama

**Affiliations:** 1 Linus Biotechnology Inc., New York, NY 10013, USA; 2 Environmental Medicine and Public Health, Mount Sinai School of Medicine, New York, NY 10029, USA; 3 Seaver Autism Center, Department of Psychiatry, Mount Sinai School of Medicine, New York, NY 10029, USA; 4 Exposure Dynamics Research Section, Health and Environmental Risk Division, National Institute for Environmental Studies, Tsukuba 305-8506, Japan; 5 Department of Pediatrics, Mount Sinai School of Medicine, New York, NY 10029, USA; 6 Center of Neurodevelopmental Disorders (KIND), Centre for Psychiatry Research, Department of Women’s and Children’s Health, Karolinska Institutet and Stockholm Health Care Services, Region Stockholm, 11330 Stockholm, Sweden; 7 Department of Medical Sciences, Child and Adolescent Psychiatry Unit, Uppsala University, 75185 Uppsala, Sweden; 8 Child and Adolescent Psychiatry, Stockholm Health Care Services, Region Stockholm, 11861 Stockholm, Sweden; 9 Curtin Autism Research Group, Curtin School of Allied Health, Curtin University, Perth, WA 6102, Australia

**Keywords:** biomarkers, autism spectrum disorder, exposomics, environmental exposures, metal exposures, diagnostic testing, neurodevelopmental disorders, hair assays, prognostic testing, dynamical methods

## Abstract

Autism spectrum disorder (ASD) is a neurodevelopmental condition diagnosed in approximately 2% of children. Reliance on the emergence of clinically observable behavioral patterns only delays the mean age of diagnosis to approximately 4 years. However, neural pathways critical to language and social functions develop during infancy, and current diagnostic protocols miss the age when therapy would be most effective. We developed non-invasive ASD biomarkers using mass spectrometry analyses of elemental metabolism in single hair strands, coupled with machine learning. We undertook a national prospective study in Japan, where hair samples were collected at 1 month and clinical diagnosis was undertaken at 4 years. Next, we analyzed a national sample of Swedish twins and, in our third study, participants from a specialist ASD center in the US. In a blinded analysis, a predictive algorithm detected ASD risk as early as 1 month with 96.4% sensitivity, 75.4% specificity, and 81.4% accuracy (*n* = 486; 175 cases). These findings emphasize that the dynamics in elemental metabolism are systemically dysregulated in autism, and these signatures can be detected and leveraged in hair samples to predict the emergence of ASD as early as 1 month of age.

## 1. Introduction

Autism spectrum disorder (ASD) is defined by persistent alterations in social communication and interactions alongside restricted, repetitive patterns of behaviors and hyper- and hyposensitivity. ASD is associated with significant impairment in social, occupational, or other important areas of adaptive functioning [1]. Co-occurrence with other neurodevelopmental conditions is common, including attention deficit hyperactivity disorder (ADHD), affecting up to 30% of children diagnosed with ASD [2]. The US Centers for Disease Control and Prevention (CDC) has reported that the average age of ASD diagnosis in the US is 4 years and 4 months [3]. These results are consistent with global data; a recent meta-analysis (55 cohorts from 35 countries, *n*  =  66,966 individuals with ASD) found a mean age at diagnosis of 60.48 months (range: 30.90–234.57 months) [4]. Neural circuits subserving the development of language and related social and sensory pathways including vision and hearing are highly plastic, especially during the first year after birth [5], but the lack of diagnostic tools and biomarkers that can be applied in infancy, prior to the development of behavioral phenotypes, are major challenges facing ASD therapy development and early intervention delivery [6]. Several randomized trials have reported cognitive and functional gains if therapy is delivered earlier in life [7,8,9,10]. There are also substantial economic benefits to early intervention on ASD, with recent estimates suggesting cost savings of 37 billion USD per year in the US alone [11]. However, in the absence of diagnostic biomarkers approved by European, US, and other international regulatory agencies that can detect the likelihood of ASD risk in the first year of life, it is not possible to start intervention during the most appropriate critical window of neurodevelopment. Purely genetic approaches have identified a broad range of genetic loci associated with ASD, but providing a diagnosis has been challenging, because genetic perturbations show marked heterogeneity within ASD cases. Here, we present a hair-based assay that leverages a sequential exposomics platform to detect risk of developing ASD as early as 1 month of age [12].

Fetal and infant exposure to toxic metals and deficiencies of nutritional elements have been linked with increased likelihood of ASD and several adverse developmental outcomes frequently associated with ASD, including intellectual disability, and language, attentional, and behavioral problems [13,14,15]. Animal studies show that the effects of various metals on brain development could be mediated through alterations in the regulation of neurotransmission, and altered frontal and subcortical brain structures [16], several of which have also been implicated in ASD [13]. Furthermore, at the cellular level, studies in animal model systems indicate that essential elements integrated through dietary sources provide critical mediation of receptor functionality in pathways related to autism-like behaviors [14,15]. Therefore, environmental and dietary exposure to metals and metal metabolism are potentially important etiological factors in ASD [13,17,18]. 

Of relevance to the studies we report here, we previously showed that the dysregulation of the dynamics underlying the metabolism of essential and toxic elements is a critical component of ASD etiology [17,18]. That work used mass spectrometry analyses of growth increments (growth rings) in teeth to generate a longitudinal profile of elemental biomarkers, thereby allowing the analysis of temporal patterns indicative of elemental metabolism to be conducted. For example, through the use of non-linear analytical methods, particularly the recurrence quantification analysis (RQA) and the cross-recurrence quantification analysis (CRQA), those studies identified patterns in elemental biomarkers indicative of periodic cycles, wherein biomarkers oscillated at predictable intervals and also experienced stable states wherein biomarker variability was minimal. These patterns were dysregulated in ASD, allowing for the development of an effective predictive algorithm to be developed utilizing the RQA/CRQA-based metrics of elemental biomarkers in teeth.

**Figure 1:**
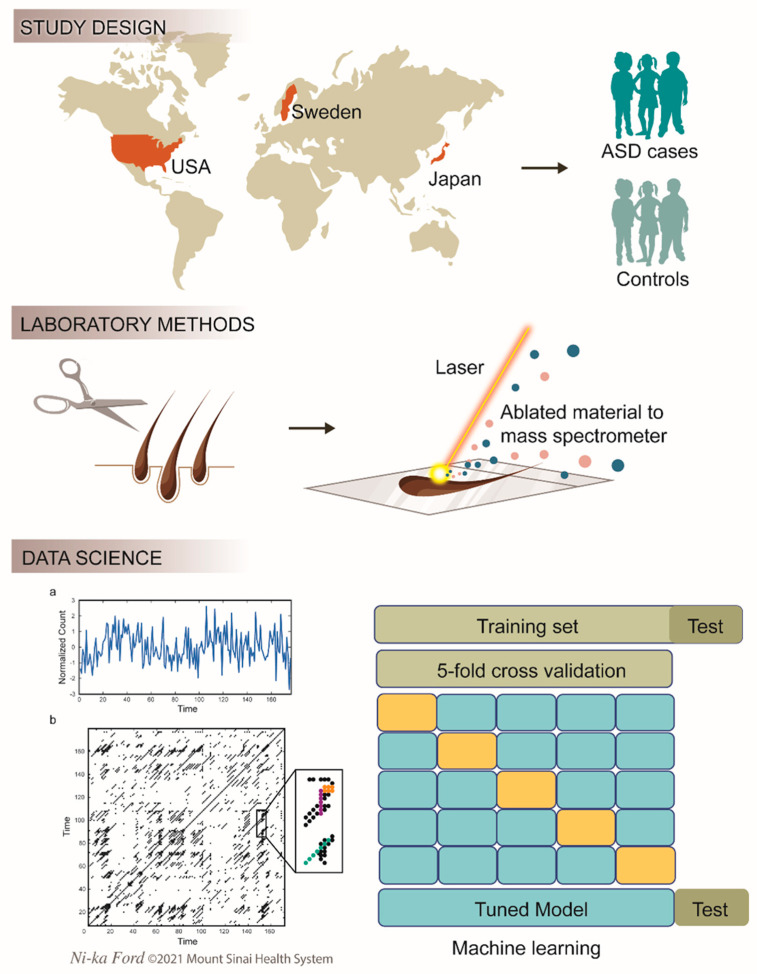
Study design and analytical pipeline. (**a**) Participants were recruited in Japan (prospective national study), Sweden (cross-sectional national study), and New York (cross-sectional, single-clinical-center study). Clinical case ascertainment was undertaken using DSM-5 criteria (Autism Spectrum Disorder, ASD). Scalp hair strands were analyzed using laser ablation-inductively coupled plasma-mass spectrometry to generate 4 to 6 hourly profiles of elemental uptake. (**b**) Recurrence and cross-recurrence quantification analyses were used to quantify the dynamic nature of elemental assimilation. A machine learning algorithm was trained on 80% of the data and tested on a randomly selected 20% holdout set.

The utility of deciduous teeth as a biomarker of ASD, however, is limited, as they are not readily accessible in the first year of life, which is why we developed a biomarker utilizing scalp hair. Specifically, for the study we present here, we used a laser ablation-inductively coupled plasma-mass spectrometry analysis of single hair strands to capture the temporal dynamics of metal metabolism. Hair strands grow in an incremental manner at a rate of approximately 1 cm per month, with some variation by age, gender, race, and growth cycle [19]. By rastering a laser along the length of the hair strand and analyzing the ablated material with a mass spectrometer, we generated 4–6 hourly sequential profiles of metal uptake for every participant (Figure 1). Critically, this approach provides indicators of both pharmacodynamic environmental inputs to the developing nervous system and pharmacokinetic processes involved in elemental metabolism. 

## 2. Materials and Methods

### 2.1. Study Populations 

Our participants were recruited from four different studies being undertaken in three countries. Ethics clearances were obtained from the relevant institutional review boards at the coordinating site of each study. The key characteristics of the studies are outlined in Table 1. 

Japan Environment and Children’s Study. This national Japanese study, also known as JECS, is a nationwide birth cohort study investigating environmental factors that might affect children’s health and development [20,21]. Fifteen Regional Centers located throughout Japan were responsible for recruiting women in early pregnancy living in their respective recruitment areas. Self-administered questionnaires and medical records were used to obtain information such as demographic factors, lifestyle, socioeconomic status, environmental exposure, medical history, and delivery information. In the period up to delivery, we collected bio-specimens, including hair.

The total number of pregnancies resulting in delivery was 100,778, of which 51,402 (51.0%) involved the program participation of male partners. Discounting pregnancies by the same woman, the study included 95,248 unique mothers and 49,189 unique fathers. The 100,778 pregnancies involved a total of 101,779 fetuses and resulted in 100,148 live births. The coverage of children in 2013 (the number of live births registered in JECS divided by the number of all live births within the study areas) was approximately 45%. Nevertheless, the data on the characteristics of the mothers and children we studied showed marked similarity to those obtained from Japan’s 2013 Vital Statistics Survey.

We selected 220 participants randomly from the 82,413 who responded to the 3-year-old questionnaire (JECS dataset, jecs-ta-20190930). The random selection was based on the following criteria: (1) Hair sample collected at 1 month of age and 1 strand available for the analyses. (2) Case selection: Randomly selected from those with confirmed autism spectrum disorder diagnosis (*n* = 372). (3) Control selection: Randomly selected to match cases by age (within same year), gender, and province (location) (*n* = 82,041).

Hair was collected from children at the age of 1 month, stored in ziplock storage plastic bags with alphanumeric barcodes and housed at the JECS biospecimen repository after being labeled. JECS participants were evaluated at 6-monthly to half-yearly intervals. At age 4, neurodevelopmental assessments were recorded, including a confirmed ASD diagnosis from a health care provider. Subsequently, to confirm the accuracy of the diagnosis, the medical record of each participant abstracted by a board-certified pediatric psychiatrist and a DSM-5 criteria diagnosis of ASD was confirmed. 

Roots of Autism and ADHD Twin Study (RATSS), Sweden. Participating twins in this study were part of RATSS, recruited between 2011 and 2016 [22]. The study was approved by Swedish Regional Ethical Review Board, and all participants gave written informed consent. Potential twin participants for RATSS are identified through nationwide registries, including Child and Adolescent Twin Study in Sweden (CATSS) [23], a population-based study of all twins born in Sweden since 1992 in which all twins are screened at age nine using Autism, Tics, ADHD and other disorders using Comorbidities Inventory (A-TAC). Participants are identified through linking Swedish Twin Registry to other national registries, such as Swedish National Patient Register, and regional clinical registers in Stockholm County (Child and Adolescent Psychiatry (“Pastill”), Habilitation & Health Centers) that include ICD-10 diagnostic information [24,25]. Finally, potential participants are also identified through Swedish societies for neurodevelopmental disorders as well as advertisements and summons in the media. Even though recruitment is performed through different routes, >80% of the twins in RATSS are present in the Swedish twin registries.

Twin pairs are recruited into RATSS either based on discordance for ASD (>2 points differences on the A-TAC autism subscale equaling ~1 SD); concordance for ASD (both twins reaching cut-off on the A-TAC autism scale); or concordance for no NDD (both twins under cut-offs for all NDD subscale on A-TAC). For other sources of recruitment, the twins are invited if at least one twin has an ICD-10 diagnosis of autism (F84.0), Asperger syndrome (F84.5), atypical autism/pervasive developmental disorder not otherwise specified (PDD-NOS) (F84.1, F84.8, and F84.9), or a Diagnostic and Statistical Manual of Mental Disorders Fifth Edition (DSM-5) diagnosis of ASD (reported by either a parent or on the registry). All potential participants undergo a telephone interview conducted by a research nurse checking eligibility before the invitation for assessment in RATSS. ASD is diagnosed according to DSM-5 criteria during a 2½-day visit at a clinical research unit based on clinical experts’ consensus and corroborated by results obtained with first-choice standardized diagnostic tools such as Autism Diagnostic Interview—Revised (ADI-R) and Autism Diagnostic Observation Schedule Second Edition (ADOS-2). Zygosity is determined by the genotyping of saliva or whole-blood-derived DNA using standard methods. Genotyping is performed using an Infinium Human-CoreExome chip (Illumina Inc., San Diego, CA, USA). The estimation of identity by descent is performed using PLINK software (v1.07) [26] after quality control and removal of SNPs with a minor allele frequency of less than 0.05 within the samples. All pairs of DNA samples showing ≥ 0.99 are considered as monozygotic pairs. Medical history and sociodemographic information of the families are collected. 

Seaver Autism Center and PRISM Study, Mount Sinai Hospital, New York, NY, USA. Seaver Autism Center is located at Icahn School of Medicine at Mount Sinai in New York City and serves a diverse and complex patient population. Seaver Center has longstanding community ties and receives approximately 600 new autism referrals annually for research and/or clinical services. Ethical approval for the study was obtained from the Mount Sinai School of Medicine research ethics committee. All participants and/or their parents provided written informed consent. In 2016, we contacted all families in ongoing studies and services at Seaver Center that [27] had a child with ASD as well as an additional child without a diagnosis of ASD. Informed consent was obtained from all parents or legal guardians. ASD diagnosis was confirmed through a gold-standard evaluation including ADOS-2, ADI-R, and a clinical evaluation with a board-certified child and adolescent psychiatrist or licensed clinical psychologist to assess DSM-5 criteria for ASD. We supplemented this sample with population-based controls from the PRogramming of Intergenerational Stress Mechanisms (PRISM) study that enrolls children of mothers receiving prenatal care from Mount Sinai Hospital. 

### 2.2. Laboratory Methods

Single hair strands from each participant were washed to remove surface contaminants in a solution of 1% Triton X-100 and ultra-pure water (18.2 MΩcm^−1^) using sonication for 1 min. Hairs were then rinsed with ultra-pure water to remove the surfactant and dried in an oven at 60 °C overnight. Hairs were mounted on plain glass microscope slides using double-sided tape and loaded into an ablation cell. A New Wave Research NWR-193 (ESI, Beaverton, OR, USA) laser ablation unit equipped with a 193 nm ArF excimer laser was connected to Agilent Technologies 8800 triple-quad ICP-MS (Agilent Technologies, Santa Clara, CA, USA) for elemental analyses. Helium was used as a carrier gas from the laser ablation cell and mixed with argon via a Y-piece before introduction into the ICP-MS. The system was tuned daily using NIST SRM 612 (trace elements in glass) to monitor sensitivity (maximum analyte ion counts), oxide formation (232Th16O+/232Th+, <0.3%), and fractionation (232Th+/238U+, 100 ± 5%). A pre-ablation scan at low laser energy (0.27–0.32 Jcm^−2^) was first run along the hair to remove the surface layer and reduce the contamination of the endogenous signal. The hair was scanned again along the same path at higher energy (0.50–0.55 Jcm^−2^) to collect element signal intensity along the strand. A path of approximately 10 mm was scanned along each hair, representing about 1 month of growth, and providing over 650 sampling points. Data were analyzed as element-to-sulfur ratios (e.g., 66Zn:34S) to control for any variations in the density within a hair and between samples. 

### 2.3. Computational Methods

The computational analysis of elemental time series involved three successive phases of feature engineering, statistical analysis, and predictive modeling. The feature engineering stage involved the application of signal processing methods, particularly a recurrence quantification analysis (RQA) and a cross-recurrence quantification analysis (CRQA), to derive descriptive statistics (features) for each element and to measure the relationships among elements. The second phase of the computational analysis involved a feature-wide association analysis, conceptually similar to a genome-wide association study (GWAS), which tested for associations between measured features and the ASD diagnostic status. The final stage of analysis involved the training of a machine learning ensemble to leverage descriptive features to predict ASD case status.

### 2.4. Feature Engineering

In the feature engineering stage of processing, for each of the 15 elemental time series measured in each hair and for the pairwise interactions among the elemental time series, a recurrence matrix or a cross-recurrence matrix, respectively, was generated to reconstruct the underlying signal dynamics [28,29]. Following the approach developed in prior studies [17,18,30,31] utilizing elemental time series, the delay (τ) and embedding dimension (m) parameters involved in recurrence plot construction were determined through the minimization of mutual information and false-nearest neighbor algorithms, respectively; likewise, to facilitate cross-subject comparison, threshold functions, ε, were constrained to yield recurrence rates of 10%. From each recurrence or cross-recurrence matrix thereby derived from each sample, an array of quantitative metrics was calculated via RQAs/CRQAs; the estimation and interpretation of these features are summarized in Supplemental Table S1. The general rationale for this framework is to derive descriptive statistics that characterize temporal dynamics in elemental time series.

More specifically, the rationale for the use of RQAs/CRQAs to analyze elemental time series derives from prior studies utilizing similar approaches, as these methods offer several advantages in this application. First, the derivation of descriptive statistics (“features”) based on signal dynamics, rather than momentary signal intensity, provides a means for measuring within-signal dynamics, which previous studies have shown are consistent across populations even when populations have varying levels of concentration [31]. Second, unlike related signal decomposition techniques, such as the Fourier analysis or Wavelet transformations, the application of RQA is robust in the presence of noise, applicable in comparatively short time series (relative to Fourier/Wavelet), and robust against non-stationarity in the data [32]. Third, prior studies that have applied RQAs/CRQAs to the longitudinal analysis of essential and non-essential elements, as utilized in this device, have shown, as noted, that RQA yields robustly generalizable measures of elemental metabolism; furthermore, these parameters are highly sensitive to systemic disease states, including autism spectrum disorder [18], attention deficit hyperactivity disorder [17], and amyotrophic lateral sclerosis [30]. Relevant to ASD, specifically, two prior studies have utilized the RQA to identify the ASD-related dysregulation of elemental metabolism and have utilized RQA-based features in the analysis of longitudinal elemental exposures to generate predictive classifiers for ASD, which were highly accurate [17,18]. 

In sum, the application of the RQA to individual elemental time series and the application of the CRQA to characterize pairwise dynamics in each potential elemental pairing yield for each time series (and pairing of time series) 12 descriptive features that characterize signal dynamics, in particular, the prevalence, duration, timing, and complexity of stable and periodic states. 

### 2.5. Quality Analysis/Quality Control

The laboratory analysis of hair requires stringent quality control and quality assurance (QA/QC) protocols. We also confirmed the reproducibility of hair element measurements by analyzing two hair samples collected from the same participant at one time point and further confirmed the stability of our elemental measures by analyzing the RQA features in hair samples collected 5 years apart in a subsample. Our results indicated a high level of reproducibility (within +/−10%) for replicate measures taken at the same timepoint as well as for replicate measures taken years apart (see Supplemental Material).

### 2.6. Statistical Analysis

The inferential analysis of RQA/CRQA features derived from each elemental time series and from pairwise dependencies among elements involved the construction of discrete generalized linear models to test for associations between RQA/CRQA features and ASD diagnostic status. For each feature, a discrete linear model was constructed to test for differences in RQA/CRQA features between ASD cases and controls. Models were adjusted for child sex and age at sample collection. *p*-values associated with elemental features were corrected for multiple comparisons with false discovery rate (FDR) adjustment. Features were batch-corrected and normalized (z-scored) prior to the statistical analysis [33]; to meet the criterion of statistical significance, FDR values were required to be less than 0.05. 

### 2.7. Predictive Modeling

**Figure 2:**
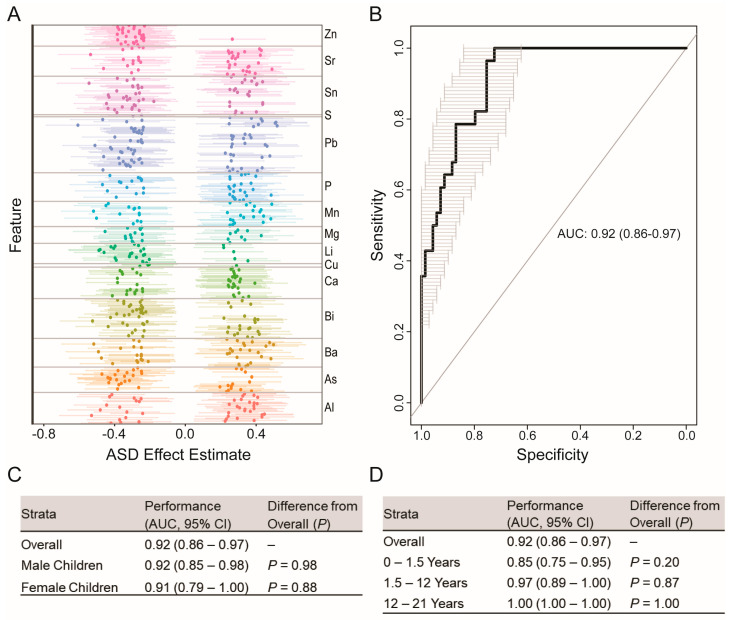
Elemental pathways associated with ASD diagnosis: (**A**) For each of 210 features measured in each elemental pathway, we constructed a discrete generalized linear model to test for associations with odds of diagnosis with ASD. Models were adjusted for age and sex, and *p*-values associated with diagnostic status were adjusted via false discovery rates (FDRs). x-axis plots the standardized effect estimate for the effect of ASD diagnosis; dots and corresponding lines reflect the effect estimate and associated 95% confidence interval for each feature. (**B**) Receiver operating characteristic [38] curve generated from predicting case status in the validation set (*n* = 97; 28 ASD). (**C**) Comparison of overall model performance with sex-stratified estimates of model performance. *p*-values reflect comparison of sex-stratified ROC curves relative to ROC curves in the overall model. (**D**) Comparison of overall model performance with age-stratified estimates of model performance. *p*-values reflect comparison of age-stratified ROC curves relative to ROC curves derived in the overall model.

The goal of predictive modeling was to utilize the descriptive statistics (“features”) generated in the descriptive analysis of the elemental time series to predict ASD case status. The model utilized for predictive classification was the form of ensemble gradient boosting introduced by Chen and Guestrin [34], referred to as XGBoost (“Extreme Gradient Boosting”). For model training, 80% of the data (*n* = 389; 147 ASD cases) were selected with random assignment. A proprietary subset of features were utilized in model training. The elemental pathways from which these features were derived are identified in Figure 2A, and additional details are provided in the supporting patent application [35]. Models were then tuned with 5-fold cross validation in the training set, with 1000 iterations of model-based optimization, per the procedure developed by Bischl et al. [36], to identify the best-performing set of hyperparameters. Following hyperparameter tuning, the selected set of hyperparameters was used to fit a model across the full set of training data. The performance of this model was then evaluated by predicting the case status in the remaining holdout dataset, comprising 20% of the total data (*n* = 97; 28 ASD), and by comparing the alignment of the predicted case status with the case status as it was assigned by the current gold-standard reference of formal clinical diagnosis of ASD according to DSM-5. 

To characterize model performance, we constructed receiver operating characteristic (ROC) curves of profile sensitivity (y-axis) and specificity (x-axis) across a range of classification thresholds. The threshold selected for performance estimation maximized Youden’s J Criterion, estimated as Sensitivity + Specificity − 1. Device performance was assessed by calculating sensitivity, specificity, and Youden’s J Criterion at the optimal classification threshold. Sensitivity was calculated as the number of Positive Cases correctly identified as Positive Cases/Total number of Positive Cases. Specificity was calculated as the number of Negative Cases correctly identified as Negative Cases/Total number of Negative Cases. 

### 2.8. Software Implementation

Feature engineering, including the implementation of RQAs/CRQAs, was performed in the Julia (v1.5.2) programming language utilizing the DynamicalSystems.jl library [37]. The visualization of recurrence matrices and some related functions were performed with Cross-Recurrence Toolbox, v5.16 (http://tocsy.pik-potsdam.de/CRPtoolbox/ accessed on 17 March 2016), in Matlab, v2019b [29]. Statistical analyses and predictive modeling were implemented in the R (v. 3.6.3) programming language. The “data.table” (v1.13.2) library was used for data manipulation; the “broom” (v0.7.1) library was used for statistical analyses and feature selection; the “pROC” (v1.16.2) and “ggplot2” (v3.3.2) libraries were used for visualizing model performance via receiver operating characteristic [38] curves; and the “mlr” (v2.19.22), “xgboost” (v1.2.0.1), and “caret” (v6.0-86) packages were used for model training, fitting, and prediction. 

## 3. Results

### 3.1. Demographic and Diagnostic Criteria

We undertook studies in three geographically distinct populations to evaluate the accuracy and generalizability of these biomarkers as predictors of ASD (*n* = 486, 175 cases; see Table 1). First, we leveraged a prospective, population-based, nation-wide study in Japan. We collected infant hair at 1 month of age, and DSM-5 clinical diagnosis for ASD was undertaken at 4 years (*n* = 220 participants and 110 ASD cases; details in Methods, Figure 1, and Table 1). Next, to account for underlying heritability, we undertook a population-based study in Sweden (*n* = 138 and 42 ASD cases). In this cross-sectional study, twin siblings were evaluated at a single clinical research center for neurodevelopmental conditions. In our third study, we collected hair from patients at a specialist autism center in the US and from a population-based study of neurotypical participants (*n* = 128 and 23 ASD cases). 

The laboratory analysis of single hair strands provided time-series measurements of 15 elements. For each individual element, we used a recurrence quantification analysis (RQA) to measure the variability in discrete elemental signals over time; we applied a complementary method, the cross-recurrence quantification analysis (CRQA), to measure cross-elemental temporal dynamics (see Methods for details). We previously applied this computational approach to time-series data of elemental uptake to quantify characteristics of metabolic dynamics, including rhythmicity, complexity, and stability [31]. Overall, this process provided 210 discrete quantitative features for each elemental pathway, accounting for the dynamics underlying the metabolism of single elements as well as pairwise dynamics across multiple elements. 

All studies undertook ASD diagnostic evaluation according to the diagnostic criterial for ASD outlined in Diagnostic and Statistical Manual for Mental Disorders, 5th edition (DSM-5), of the American Psychiatric Association. To fulfill diagnostic criteria of ASD using the DSM-5, all three symptoms of social affective difference need to be present in addition to two of four symptoms related to restrictive and repetitive behaviors [6]. We provide additional detail on the presence of intellectual, developmental, psychiatric, and genetic comorbidities in Supplemental Table S3. 

### 3.2. Elemental Signatures of ASD Phenotype

Our initial computational analysis focused on discrete associations between individual biomarkers and ASD diagnosis. In Figure 2A, we show the results of this analysis, wherein we identified 567 individual features that significantly differed between ASD cases and controls after adjustment for age and sex, and false discovery rate (FDR) correction for multiple comparisons. Two general patterns emerged from this analysis. First, our findings indicated a broad pattern of dysregulated elemental dynamics, which spanned every elemental pathway investigated. This was apparent from the representation of all 15 elements and the even spread of features with positive and negative effects, a pattern generally indicative of bidirectional effects suggestive of broad dysregulation. Second, in a subset of essential and non-essential elements, including zinc, lithium, and copper, these effects were almost entirely unidirectional, with ASD cases exhibiting attenuated periodic dynamics relative to controls. These findings agree with earlier epidemiologic reports that showed systemic changes in the regulation of elements in ASD [17,18,39], particularly zinc and copper, and are supported by mechanistic findings in animal models [40,41].

### 3.3. Prediction of ASD Case Status

Next, we sought to leverage these patterns in the construction of an ensemble model to predict ASD case status. The data were combined from all populations and randomly portioned in a training set (80% of data; *n* = 389, 147 ASD cases) for model training, and a validation set (*n* = 97; 28 ASD cases) to evaluate model performance. Figure 2B shows the receiver operator characteristic [38] curve derived from predicting ASD case status in the validation set. According to the optimal criterion, this model yielded 96% (95% I: 82–100%) sensitivity, 75% (95% CI: 64–85%) specificity, and 81% (95% CI: 72–89%) overall accuracy. Youden’s index (J), a summary statistic of the ROC curve [42], was equal to 0.71. A full confusion matrix is provided in Supplemental Table S2. 

We also considered how device performance varied across key demographic and biological factors, including age and sex. In Figure 2C, we provide sex-stratified indicators of device performance for participants in the holdout validation dataset. In general, sex-stratified model performance, summarized as an AUC, did not differ from overall model performance for male (*p* = 0.98) and female (*p* = 0.88) participants. Likewise, as shown in Figure 2D, when stratifying participants in age categories relative to early life, pre-adolescence, and adolescence, we found that age-specific estimates of device performance did not significantly differ from overall device performance. 

## 4. Discussion

It is desirable to identify objective biomarkers that can predict the emergence of ASD before the development of behavioral symptoms and support early intervention. Current efforts to achieve these goals have focused on genomic loci, but multiple lines of evidence consistently implicate environmental associations with the etiology of ASD [13,43]. Here, we developed a novel analytical paradigm that leverages high-resolution longitudinal sampling to measure temporal dynamics in elemental uptake and metabolism, thereby leveraging signatures of both the internal and external environment. This approach allowed a model to be generated that accurately predicts the later emergence of ASD using samples collected from children as young as 1 month of age. 

The development of this paradigm builds upon several recent studies that employed similar approaches, particularly in terms of the application of RQAs/CRQAs to characterize temporal dynamics in elemental biomarkers. In previous work, we identified signatures in elemental metabolism that were highly predictive of ASD status [18]; in particular, we found that the prevalence, duration, and complexity of cyclical processes in elemental metabolism were reduced in ASD cases. However, though that study’s analytical methods were largely similar to those employed here, it did not include prospectively collected samples, and the tissue matrix investigated (teeth) is not accessible soon after birth as is hair. Likewise, a related study showed that temporal dynamics in elemental metabolism, measured as in the present study via the application of RQAs/CRQAs, were effective in discriminating samples collected from neurotypical children and from those with ASD, ADHD, or comorbid ASD/ADHD diagnoses [17]. In extending these methods to the analysis of hair samples and applying this approach to the analysis of prospectively collected samples, the present study thus builds upon prior established work to generate a novel hair-based ASD biomarker.

Both the strengths and limitations must be considered when interpreting the results of this study. The ethnically and geographically diverse study populations assessed here support broad biological generalizability, but future studies are needed to replicate and refine the predictive algorithms generated here to develop an effective medical diagnostic device. The prevalence of ASD in our study population was 28%. This is substantially higher than the 2–3% prevalence in the general population of children but closer to the prevalence observed in groups with a high likelihood of ASD. For example, based on the estimates of Hansen and colleagues, who studied 2.5 million births in six countries for the risk of ASD when an older sibling had an ASD diagnosis, the prevalence estimates was between 9% and 17% when a full sibling has ASD [44] and even higher when multiple risk factors co-exist (for example, 29% of children with ADHD also had ASD [45]). Advancing paternal and maternal age have both been associated with ASD. Specifically, for fathers 45 years or older, the relative risk of having a child with ASD is 30–50% higher than fathers who are 20–29 years of age [46]. The increase in maternal age and a mismatch in maternal and paternal age are also associated with an increased risk of having a child with ASD [46,47]. Infants and children with ASD are known to fall behind on developmental milestones [48]. Several clinical tools are available, such as Modified-CHecklist for Autism in Toddler (M-CHAT), First Years Inventory (FYI), and Quantitative-CHecklist for Autism in Toddler (Q-CHAT), which may be used by clinicians to evaluate children. However, they have low-to-moderate accuracy when used alone and would benefit from additional information provided by a biomarker-based diagnostic aid [38]. Overall, the biomarker we propose here must be viewed as a diagnostic aid that can assist in the early detection of ASD in conjunction with a thorough clinical evaluation.

One limitation in the current study, a topic which should be the focus of future studies, is the stability of biomarker measurements over the course of long developmental time windows. Ideally, this will be addressed in a longitudinal study to quantify the extent to which a given elemental biomarker varies in the same individual from the early postnatal period to later childhood maturation. This is particularly important in comparison to the design of the current study, which characterized biomarkers across a broad age range in childhood cross-sectionally but did not analyze biomarker stability in the same individual over time. However, the fact that the same biomarker was consistently accurate in early infancy (our youngest participants were one month old) and also in early adulthood (our oldest participants were 21 years) supports the stability of the ASD biomarker shown here. Another limitation implicit to this sort of tool is that the device is entrained to align with current diagnostic standards—in this case, a binary (positive/negative) diagnosis for ASD via the DSM-V. One implication of this is that the device will implicitly “inherit” any shortcomings in the diagnostic standard to which it is entrained. In particular, given that ASD is considered a spectrum of symptoms and may involve multiple phenotypes, the device will need to be updated and retrained as gold-standard diagnostic criteria emerge to identify ASD subtypes, as opposed to the dichotomous diagnosis entrained here. Similarly, future studies are planned to entrain a device to measures of symptom severity, in order to better capture the spectrum of symptoms associated with ASD.

The sensitivity and specificity estimates we report here must be considered in relation to the background prevalence of ASD in the enriched-risk populations where the hair biomarker could appropriately be used. The NPV of the device in this diverse sample population suggests that this approach can effectively provide negative diagnostic indicators in diverse clinical conditions with varying disease prevalence; however, positive diagnostic indicators will need to be cautiously interpreted and will decline in utility as the device is applied to broader populations where prevalence is reduced. One potential solution to this challenge would be the extension of the single-sample method applied here to the evaluation of multiple samples. By analyzing three hairs in tandem, for example and requiring three consistent “positive” results to serve as criterion for positive diagnosis, PPV could be increased to 94%, assuming other factors remain constant. Future studies are needed to evaluate the potential benefits of such an ensemble and, more generally, to validate and confirm that these features provide stable diagnostic performance across larger and more diverse populations. Likewise, the stability of the features measured here should be assessed through repeat prospective sampling over the course of the lifespan, and future studies should evaluate the efficacy of this approach in predicting ASD in adults. We, however, show that the elemental dynamics in hair that are relevant to ASD diagnosis are highly stable using repeat samples collected from the same participants 5 years apart (see Supplemental Material). Of note, our analysis of hair focuses on the dynamics of elemental metabolism as measured in hair and is not reliant on concentrations of metals in hair. The integration of a prospective study design, wherein samples were collected soon after birth, prior to the development of symptoms and later diagnosis, is a particular strength of this study that future studies should emulate. 

## 5. Conclusions

Overall, we report a novel integrated platform that combines the high-resolution longitudinal sampling of essential, non-essential, and toxic elements in hair with a machine learning ensemble for the prediction of ASD case status. Applying computational methods derived from chaos theory, particularly the RQA and the CRQA, we characterized single- and across-element temporal dynamics that underlie ASD. Our approach contrasts with previous efforts that relied solely on genetic readouts to identify ASD biomarkers. Instead, we focused on the interface of genetically driven metabolic signatures with external environmental exposures, thus considering both the genetic origins and the environmental triggers that lead to ASD. The biomarker we propose here establishes the potential to develop a diagnostic aid that may help to improve the lives of ASD-affected individuals with diagnosis at a younger age that facilitates early intervention.

## 6. Patents

Patents resulting from this work include “Systems and methods for diagnostics for biological disorders associated with periodic variations in metal metabolism” [35].

## Figures and Tables

**Table 1:** Participant characteristics.

Study	Location	Design	N (Cases)	Male/Female	Age, Months (SD)
JECS	Japan	National prospective study	220 (110)	110/110	1 (0)
RATSS	Sweden	National cross-sectional study	138 (42)	76/62	170.8 (36.9)
Seaver	USA	Cross-sectional study	128 (23)	80/48	61.6 (33.4)

JECS, Japan Environment and Children’s Study. RATSS, Roots of Autism and ADHD Twin Study in Sweden. Seaver, Seaver Autism Center Study. Reported age indicates participant age at sample collection.

## Data Availability

Data generated in this study may include personal health information, and as such, all reasonable requests must be evaluated by appropriate ethical review committees at the involved institutions.

## Supplementary Materials

The following supporting information can be downloaded at: https://www.mdpi.com/article/10.3390/jcm11237154/s1, Figure S1: Average percent difference across elements in Shannon entropy, Figure S2: Average percent difference across elements in determinism, Figure S3: Recurrence analysis of elemental data derived from hair, Table S1: Features derived from recurrence and/or cross-recurrence quantification analyses of elemental time-series data, Table S2: Confusion matrix: frequencies of predicted diagnoses and actual diagnoses, Table S3: Comorbid diagnosis in participants with and without positive diagnosis or ASD.
