# Supplementary material for: Elemental Dynamics in Hair Accurately Predict Future Autism Spectrum Disorder Diagnosis: An International Multi-Center Study
Source: J Clin Med. 2022 Dec 1;11(23):7154. doi: 10.3390/jcm11237154 (PMC9740182; doi:10.3390/jcm11237154)
Supplement: Supplementary file 1 [file jcm-11-07154-s001.zip › jcm-2019708-supplementary.pdf]

Supplement for Elemental dynamics in hair accurately predict future autism spectrum disorder diagnosis: an international multi-center study

Christine Austin[1,2†], Paul Curtin[1,2†*], Manish Arora[1,2†*], Abraham Reichenberg[1,3†], Austen Curtin[1,2†], Miyuki Iwai-Shimada[4], Robert Wright[1], Rosalind Wright[1,5], Karl Lundin Remnelius[6], Johan Isaksson[6,9], Sven Bölte[6,7,8], Shoji F. Nakayama[4]

1 Linus Biotechnology Inc., New York, NY 10013, USA
2 Environmental Medicine and Public Health, Mount Sinai School of Medicine, New York, NY 10029, USA
3 Seaver Autism Center, Department of Psychiatry, Mount Sinai School of Medicine, New York, NY 10029, USA
4 Exposure Dynamics Research Section, Health and Environmental Risk Division, National Institute for Environmental Studies, Tsukuba 305-8506, Japan
5 Department of Pediatrics, Mount Sinai School of Medicine, New York, NY 10029, USA
6 Center of Neurodevelopmental Disorders (KIND), Centre for Psychiatry Research, Department of Women's and Children's Health, Karolinska Institutet and Stockholm Health Care Services, Region Stockholm, 11330 Stockholm, Sweden
7 Department of Medical Sciences, Child and Adolescent Psychiatry Unit, Uppsala University, 75185 Uppsala, Sweden
8 Child and Adolescent Psychiatry, Stockholm Health Care Services, Region Stockholm, 11861 Stockholm, Sweden
9 Curtin Autism Research Group, Curtin School of Allied Health, Curtin University, Perth, WA 6102, Australia
* Correspondence: paul.curtin@linusbio.com (P.C.); manish.arora@mssm.edu (M.A.)
† These authors contributed equally to this work.

Index

1. Quality Assurance and Quality Control Procedures for Laboratory Hair Analysis
2. Derivation of Descriptive Features
3. Model Performance – Confusion Matrix
4. Participant Comorbidity Characteristics

I. QA/QC for Laboratory Analysis of Hair Samples

Reproducibility of results

A second hair strand was analyzed for a subset of 20 samples for QA/QC. Element signal profiles from duplicates are aligned and the percent difference between each sequential data point measured and averaged. A difference of < ± 20% (recovery 100 ± 20%) was considered acceptable. Percent difference between duplicates is typically less than 10% for most elements (see Figure S1).

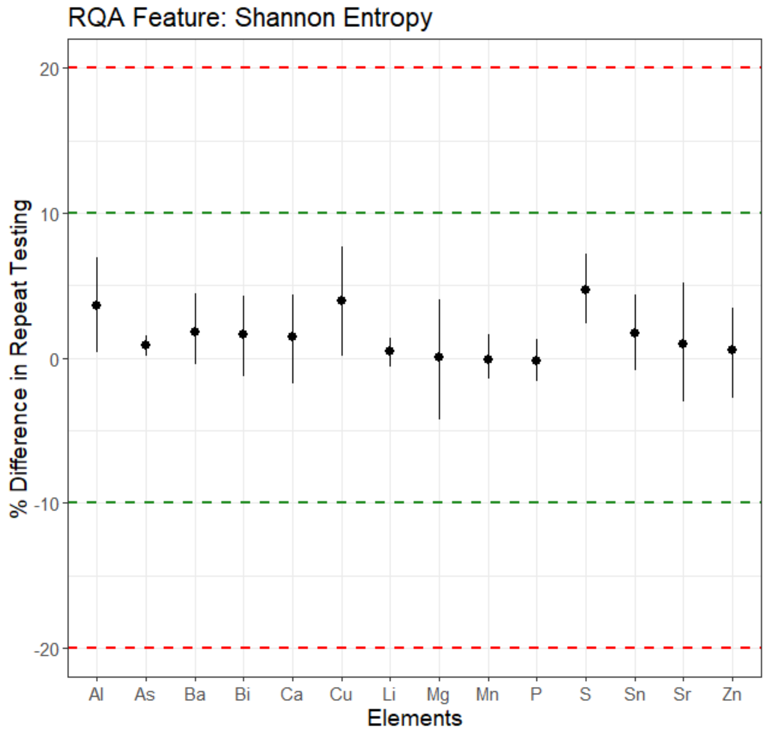

Figure S1. Average percent difference across elements in bioinformatic features – here, Shannon Entropy, estimated via Recurrence Quantification Analysis – measured in two hairs collected from the same individual at the same time but analyzed by LA-ICP-MS months apart. Green dashed line indicates ± 10% difference between duplicates; red dashed line indicates +/- 20% difference between duplicates. n=20 participants.

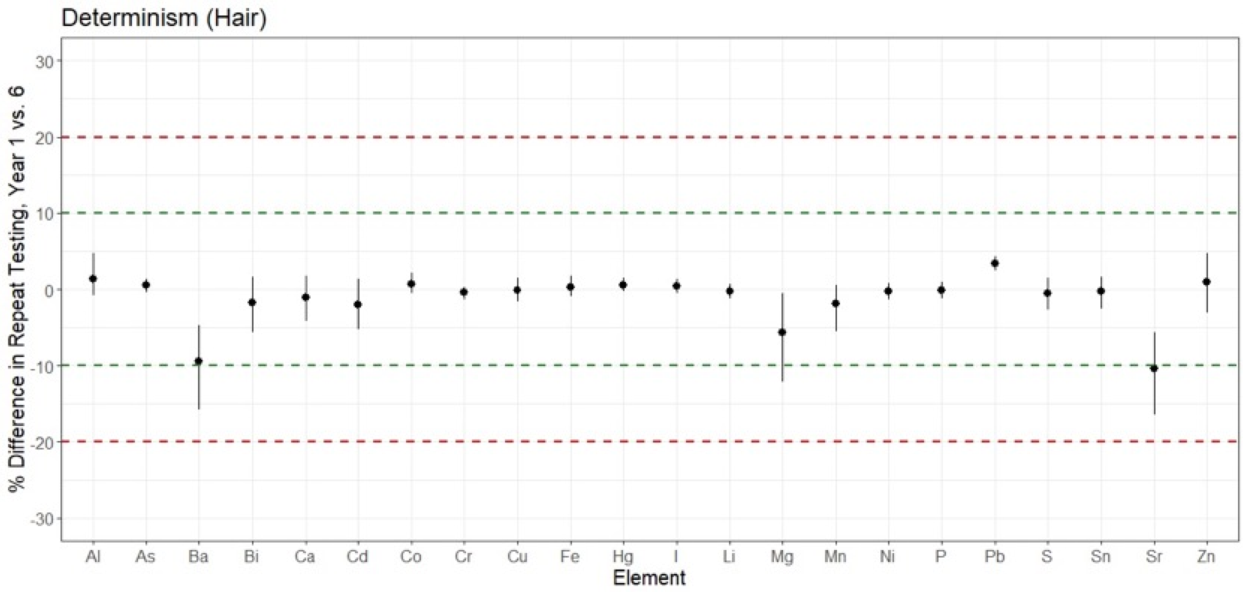

Figure S2. Average percent difference across elements in bioinformatic features – here, Determinism, estimated via Recurrence Quantification Analysis – measured in two hairs collected from the same individual 5 years apart (at age 1 year and then at age 6 years). Green dashed line indicates ± 10% difference between duplicates; red dashed line indicates +/- 20% difference between duplicates. n=20 participants.

II. Derivation of Descriptive Features

For each of 15 elemental time series measured in each hair, and for the pairwise interactions between each elemental time series, a recurrence matrix or cross-recurrence matrix, respectively, was generated to reconstruct underlying signal dynamics. Supplementary Figure S3 provides an example of this process in the analysis of a single hair trace. The formation of diagonal lines in a recurrence matrix indicates the formation and dissolution of transient periodic processes. Likewise, the emergence of vertical and horizontal lines, referred to as laminar structures, indicates periods of relative signal stability. Recurrence quantification analysis (RQA) involves the quantitation of these features to characterize the magnitude and prevalence of these features in different signals; likewise, cross-recurrence quantification analysis (CRQA) extends this approach to consider pairwise dependencies between signals. From each recurrence and cross-recurrence matrix derived from each sample, quantitative metrics are derived, as described in accompanying Table S1.

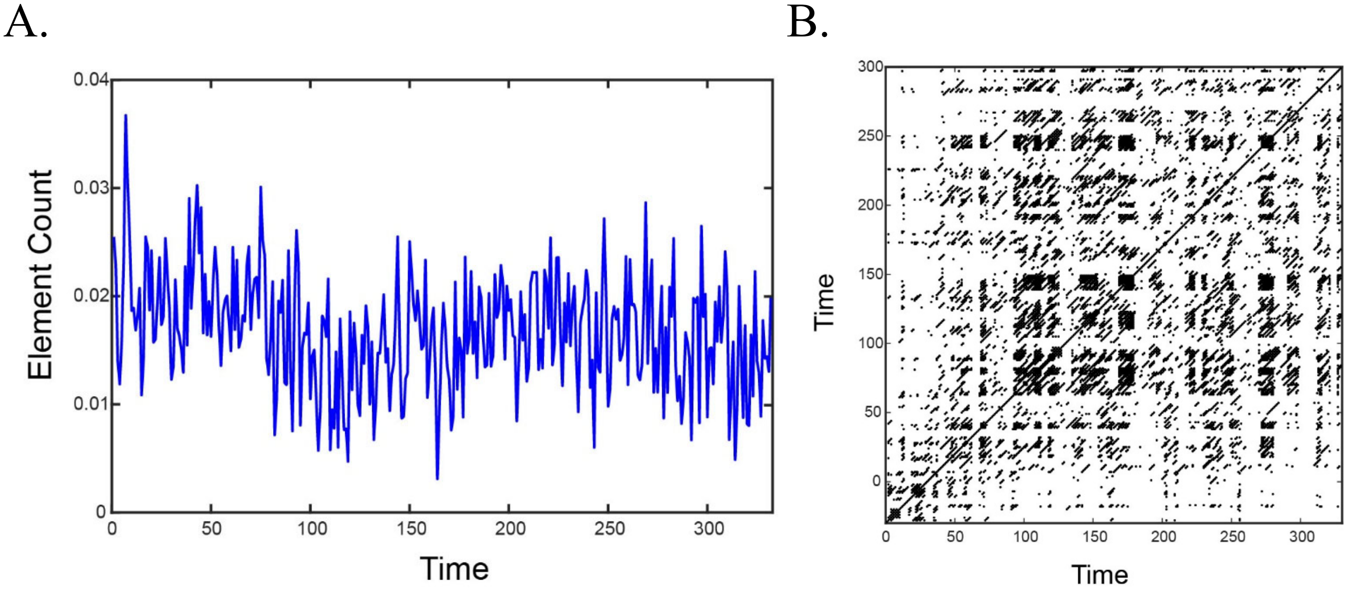

Figure S3. Recurrence analysis of elemental data derived from hair. In left panel, (A), elemental (Phosphorus) time-series data extracted from analysis of a hair sample are shown. In right panel, (B), a recurrence matrix is shown. This formation of diagonal and vertical lines in the recurrence matrix is indicative of underlying periodic dynamics in the time series. In recurrence quantification analysis, the distribution of these features is quantified to derive metrics descriptive of underlying processes in the elemental time series.

Table S1. Features derived from recurrence and/or cross-recurrence quantification analysis of elemental time series data.

Feature	Algorithm	Interpretation
Recurrence Rate (RR)	$$RR = \frac{1}{N-k}\sum_{j-1=k}^{N-k} R(i,j)$$ Where k is the distance of a given diagonal line from the central diagonal in a square recurrence or cross recurrence matrix, N is the total number of diagonal processes, and *R(i,j)* is the probability of recurrence at a given coordinate *i,j*.	This feature measures the ratio of recurrent points, typically indicated in black, vs. non-recurrent points (white space) in a recurrence matrix or cross-recurrence matrix. This measure reflects the incidence of repetition in each signal. In these analyses, threshold functions were set adaptively to yield an RR=0.1 in all analyses; as RR was therefore a constant, this feature was excluded from analysis.
Determinism (DET)	$$DET = \frac{\sum_{l=lmin} lP(l)}{\sum R}$$ Where *l* is the length of diagonal lines in the recurrence matrix, *P(l)* is the number of lines with length equal to *l*, and *R* is the number of recurrence points in the full recurrence plot.	This feature measures the ratio of diagonal points to non-diagonal points in a recurrence matrix / cross-recurrence matrix. This reflects the prevalence of periodic processes in the signal.
Mean Diagonal Length (MDL)	$$MDL = \frac{\sum_{l=l_{min}}^{N} lP(l)}{\sum_{l=l_{min}}^{N} P(l)}$$ Where *N* is the number of diagonal lines, l_{min} is the minimum specified length of diagonal lines (in this application, 2), *l* is the length of a given diagonal line, and *P(l)* is the histogram of all diagonal lines.	This feature measures the average length of diagonal lines in a recurrence matrix / cross-recurrence matrix. This measure reflects the duration of periodic processes in the signal.
Maximum Diagonal Length (LMAX)	$$LMAX = \max(P(l))$$ Where *P(l)* is the histogram of diagonal lines in a recurrence or cross recurrence matrix.	This feature measures the longest diagonal line in a recurrence matrix / cross-recurrence matrix. This measure reflects the duration of the most persistent periodic process.
Shannon Entropy in Diagonal Length (ENT)	$$ENT = -\sum_{l=l_{min}}^{N} p(l)\ln\ p(l)$$ Where *l* indicates the length of a diagonal line, l_{min} indicates the minimum length of diagonal lines (in this application, 2), *N* is the total	In a recurrence matrix / cross-recurrence matrix, this feature measures the predictability of the distribution of diagonal line length. This can be interpreted as a measure of complexity involved in periodic processes in the signal.

	number of diagonal lines, and *p(l)* is the probability distribution of diagonal lines.	
Trend in Recurrences (TREND)	$$TREND = \frac{\sum_{i=1}^{N}(i-\frac{N}{2})(RR_i - \langle RR_i \rangle)}{\sum_{i=1}^{N}(i-\frac{N}{2})^2}$$ Where N is the number of diagonal lines parallel to the maximal diagonal in a square recurrence or cross-recurrence matrix, i is a diagonal line, RR_i is the diagonal-wise recurrence rate, and $\langle RR_i \rangle$ is the average diagonal recurrence rate.	This feature measures the relative density or sparseness of a recurrence matrix at the matrix edges relative to the center. This measure reflects the trend in the signal to depart from stationarity.
Laminarity (LAM)	$$LAM = \frac{\sum_{v=l_{min}} vP(v)}{\sum R}$$ Where v is the length of vertical lines the recurrence or cross-recurrence matrix, l_{min} is the minimum length of vertical lines (in these applications, set to 2), and $P(v)$ is the histogram of vertical line lengths. R refers to the sum of all recurrence points; as such, this function essentially constructs a ratio of recurrence points which contribute to vertical lines relative to all recurrence points.	This feature measures the ratio of vertical lines in a recurrence or cross recurrence matrix relative to points that do not comprise vertical lines. This measure characterizes the prevalence of periods of stability in a given signal.
Trapping Time (TT)	$$TT = \frac{\sum_{v=l_{min}} vP(l)}{L}$$ Where v is the length of vertical lines the recurrence or cross-recurrence matrix, l_{min} is the minimum length of vertical lines (in these applications, set to 2), and P(l) is the histogram of vertical line lengths. L in this case refers to the frequency of all vertical lines meeting l_{min} criteria; thus, in essence this measure reflects the mean vertical line length.	This feature measures the distribution of temporal intervals between vertical lines in a recurrence matrix or cross recurrence matrix. This measure provides the mean interval observed between periods of signal stability.
Maximum Vertical Line Length (VMAX)	$$VMAX = \max(P(v))$$ Where $P(v)$ indicates the distribution of vertical line lengths.	This feature measures the maximum length in the distribution of vertical line lengths observed in a recurrence matrix or cross-recurrence matrix. This measure reflects the peak duration of stable processes in a given signal.
Shannon Entropy in Vertical Line Lengths (VENT)	$$VENT = -\sum_{v=l_{min}}^{N} p(v) \ln\, p(v)$$ Where l indicates the length of a vertical line, l_{min} indicates the minimum length of vertical lines (in this application, 2), N is the total number of vertical lines, and $p(v)$ is the probability distribution of vertical lines.	This feature measures the predictability of the distribution of vertical line lengths in a recurrence or cross-recurrence matrix. This measure reflects the complexity in the distribution of intervals of signal stability.

Mean Recurrence Time (TMRP)	$TMRP = \sum_{w=1}^{N} wP(w) \Big/ \sum_{w=1}^{N} P(w)$ Where w is the length of vertical white lines, N is the total number of vertical white lines, and *P(w)* is the frequency distribution of vertical white line lengths.	This feature measures the mean duration of intervals between vertical lines indicative of signal stability in a recurrence or cross-recurrence matrix.
Shannon Entropy in Recurrence Time (WENT)	$WENT = -\sum_{w=1}^{N} p(w)\ln\ P(w)$ Where w is the length of vertical white lines, and *P(w)* is the frequency distribution of vertical white lines.	This feature measures the predictability of the distribution of intervals separating vertical lines indicative of signal stability in a recurrence or cross-recurrence matrix.
Number of the most probable recurrence time (NMPRT)	$NMPRT = \max\left(P(w)\right)$ Where P(w) indicates the histogram of the vertical white line distribution	This feature measures the maximum value of the histogram of recurrence times. This measure indicates the most probable interval between periods of signal stability.

III. Model Performance – Confusion Matrix

Table S2. Confusion matrix: frequencies of predicted diagnoses and actual diagnoses.

		Reference Test - DSM-V		
		Positive	Negative	Total
Model Predictions	Positive	27	17	44
	Negative	1	52	53
	Total	28	69	97

Table cells reflect the number of predicted diagnostic outcomes relative to actual diagnostic status as determined by the DSM-V in the holdout validation dataset.

IV. Participant Comorbidity Characteristics

Table S3. Comorbid diagnoses in participants with and without positive diagnosis for ASD.

Disorder	ASD	Control
ADHD	26	21
Intellectual	16	4
Developmental[1]	30	17
Psychiatric[2]	16	6
Genetic[3]	9	5
No comorbidity	123	279

[1]Developmental Delay, Learning Disorder, Language Disorder
[2]Anxiety, Depression, OCD, Phobia, PTSD, Tics, Tourette's
[3]Genetic Syndrome, PMS, DDX3X, FOXP1, ADNP

Cells reflect counts (n) of participants with positive diagnoses in each broad diagnostic category; note diagnoses are non-exclusive, such that a patient might receive multiple diagnoses.
